# Dietary Supplementation of Methionine, Tryptophan, and Threonine for Pigs Under Sanitary Challenges: Current Knowledge and Future Directions

**DOI:** 10.3390/vetsci12090794

**Published:** 2025-08-23

**Authors:** Graziela da Cunha Valini, Alícia Zem Fraga, Ismael França, Danilo Alves Marçal, Pedro Righetti Arnaut, Alini Mari Veira, Marllon José Karpeggiane de Oliveira, Ines Andretta, Luciano Hauschild

**Affiliations:** 1 Department of Animal Science, School of Agricultural and Veterinarian Sciences, São Paulo State University (Unesp), Jaboticabal 14884-900, Brazil; graziela.valini@unesp.br (G.d.C.V.); ismael.franca@unesp.br (I.F.); pedro.arnaut@unesp.br (P.R.A.); alini.mari@unesp.br (A.M.V.); marllonjkoliveira@hotmail.com (M.J.K.d.O.); 2 Department of Animal Science, Universidade Federal Rural do Rio de Janeiro, Seropedica 23890-000, Brazil; azfraga@ufrrj.br; 3 Graduate Program in Animal Production, Universidade Brasil, Fernandopolis 15600-000, Brazil; danilo.a.marcal@hotmail.com; 4 Department of Animal Science, Faculty of Agricultural and Life Sciences, Universidade Federal do Rio Grande do Sul, Porto Alegre 91540-000, Brazil; ines.andretta@ufrgs.br

**Keywords:** health, immune system, inflammation, nutrition, swine

## Abstract

Sanitary challenges (SCs) may alter the health status, growth performance, and pigs’ welfare. Changes in amino acid (AA) plasma concentrations have been observed in inflammatory-challenged pigs which may be associated with key factors, such as: (1) the synthesis of immune components to support innate and/or adaptive immune responses, (2) the redistribution of nutrients from growth and production functions toward cells and tissues involved in inflammatory and immune responses, and (3) decreased anabolism and/or increased catabolism of skeletal muscle to increase the availability of nutrients, often as a consequence of reduced feed intake. Due to their health-promoting effects, nutritional strategies involving AA may help mitigate the negative impacts of SC. Methionine, tryptophan, and threonine, beyond serving as protein building blocks, are considered functional AA because they support immune system function, enhance intestinal barrier integrity, modulate inflammatory responses, and limit oxidative stress. Additionally, the review highlights the influence of individual variability, such as differences in body weight, on nutritional requirements and responses to AA supplementation for pigs under SC. The integration of nutritional strategies tailored to immune-challenged pigs offers promising avenues to improve productivity and animal welfare in commercial swine production systems with increasing restrictions on antibiotic use.

## Simple Summary

Pigs raised in commercial production systems are often exposed to unsanitary conditions that impair health, welfare, and growth performance. An increase in environmental pathogenic pressure can be exacerbated when biosecurity protocols, stocking density, and the degree of hygiene of the facilities are not adequately followed. These sanitary challenges trigger immune and metabolic responses that divert nutrients (in particular, amino acids; AA) from growth to defense mechanisms. Methionine, tryptophan, and threonine are considered functional AA due to their roles in supporting immune function, antioxidant defense, and gut integrity. This review emphasizes the critical role of these AA in alleviating the detrimental effects of sanitary stress. Furthermore, the integration of functional AA supplementation with precision feeding may offer promising solutions to enhance pig resilience and sustainability in antibiotic-free production systems.

## 1. Introduction

Pig production and pork consumption play an important economic role in agricultural systems worldwide. This has been made possible through the optimization of pig performance across the different phases of the production cycle. However, the selection for traits such as weight gain and lean body mass deposition has been prioritized over traits associated with disease resistance [1,2]. Additionally, global climate change may negatively impact pig production, as high temperatures can disrupt the balance between host and environment, exposing pigs to higher microbial pressure [3], particularly in group-housed pigs raised under intensive production systems.

Pigs may face health disorders that reduce farm profitability due to impaired growth performance [4], and increased production costs related to feed, medication, and mortality [5,6]. For instance, pigs reared in conventional housing systems with high microbial loads grow 10 to 20% more slowly than pigs kept in clean’ environments [7,8], highlighting the importance of hygiene and biosecurity in maintaining animal performance. However, maintaining such controlled conditions is not always feasible in commercial settings, particularly considering growing concerns about antimicrobial resistance. While this is a positive step toward more sustainable production, restricting the use of in-feed antibiotics also presents new challenges: pigs are now more frequently exposed to subclinical infections and microbial pressure, particularly in systems with compromised hygiene or biosecurity.

Thus, there is a growing need for nutritional strategies that can support pig health and performance under challenging conditions without relying on antibiotic intervention. Among these strategies, the supplementation of amino acids (AA) has emerged as a promising approach due to their role in immune responses [9,10]. This strategy is also supported by findings that immune-challenged pigs often show reduced voluntary feed intake [11] and increased muscle catabolism to release AA for immune cell synthesis [12]. For instance, growing and finishing pigs reared under poor sanitary conditions or challenged by *Salmonella* Typhimurium (ST) have shown improved feed efficiency and weight gain when fed a diet supplemented with higher levels of methionine (Met), tryptophan (Trp), and threonine (Thr) [13,14,15]. Likewise, post-weaning piglets housed in poor hygiene conditions showed increased feed consumption when fed a diet with 17% higher Trp intake [16].

Although there is increasing evidence that AA requirements change with immune system activation, most findings have come from short-term studies involving individual (based on body weight and body composition) or small-group-housed post-weaning piglets supplemented with a single AA [17,18,19,20]. It should be noted that pigs in commercial conditions have been housed in large groups. Thus, it remains unclear to what extent these results can be extrapolated to a commercial condition where pigs are chronically exposed to multiple antigens without severe clinical signs of disease or mortality [13], and where simultaneous supplementation with more than one AA may be required. For instance, recent studies have shown that dietary supplementation of Met, Trp, and Thr 20% above National Research Council [NRC] [21] recommendations improve the performance of growing group-housed pigs under sanitary challenge [22,23]. However, this response varied by body weight categories evaluated, with lighter pigs benefiting more from AA supplementation than heavier animals [24].

As pigs are increasingly exposed to different challenging conditions, it is of utmost importance to deepen our understanding of how AA supplementation influences their adaptive responses, exploring the individual variability. Therefore, our objective with this review is to summarize and critically evaluate current scientific evidence on the effects of functional AA supplementation—specifically Met, Trp, and Thr—on the performance, immune response, and behavior of pigs under sanitary challenge conditions. It also discusses the variability in individual responses and highlights future research perspectives for applying this nutritional strategy in commercial swine production.

## 2. Metabolic and Physiological Responses of Pigs Under Sanitary Challenge Conditions

Sanitary challenges (SC), including bacterial or viral infections, parasitic infestation, and the degree of hygiene conditions, may affect pig performance and, consequently, compromise the economic outcomes of production systems. Under these conditions, pigs activate their immune system, triggering a cascade of neuroendocrine and metabolic responses aimed at restoring homeostasis [25]. Among these, the reduction in voluntary feed intake [11] and intense muscle catabolism to mobilize AA for immune cell synthesis [12] are highlighted. Even with the absence of clinical signs, SC pigs often show reduced appetite and growth compared to non-challenged counterparts [23]. Such physiological adjustment can negatively impact farm profitability. For instance, under commercial conditions, pigs under SC during the growing phase have shown reduced carcass yield and incurred economic losses of up to USD 26.10 per pig [5].

Physiological mechanisms are activated to help maintain the animal’s integrity and control pathogen proliferation. The first line of defense consists of mechanical and chemical barriers, such as skin, gastric acid, bile secretion, and mucus production [26]. For instance, pigs under SC exhibit increased production of mucus and immunoglobulins [27], which supports intestinal integrity and helps prevent pathogens’ translocation across the gut mucosa.

Under a SC, two primary defense mechanisms are activated: innate and adaptive immunity. The activation and efficiency of these mechanisms depend on the nature of the stimulus as well as the pigs’ immune and nutritional status [28,29]. The innate immune system extends beyond physical barriers and includes mononuclear phagocytes (e.g., monocytes and macrophages), dendritic cells, polymorphonuclear granulocytes (such as neutrophils, eosinophils, and basophils), mast cells, natural killer cells, platelets, and humoral factors (lysozymes, C-reactive proteins, and interferons). This system provides a rapid and non-specific response to invading pathogens and plays a critical role in triggering the adaptive immune response. As an immediate response to SC, innate immune activation is characterized by increased plasma concentrations of cytokines, glucocorticoids [3], and acute phase proteins like haptoglobin [30], along with alterations in insulin sensitivity and reduced circulating levels of thyroid hormones [31].

Another clinical sign of infection and inflammation associated with the innate response is fever. Pro-inflammatory cytokines released by phagocytes, such as IL-1, IL-6, and tumor necrosis factor-alpha (TNF-α), act as pyrogenic mediators by stimulating the synthesis of PGE2, which plays a central mediating role in triggering the febrile response [32]. The onset of fever increases the immune system’s energy demands. For a pig weighing 80 kg, each degree Celsius increase in body temperature is estimated to raise basal metabolic rate by around 10% [33].

**Figure 1:**
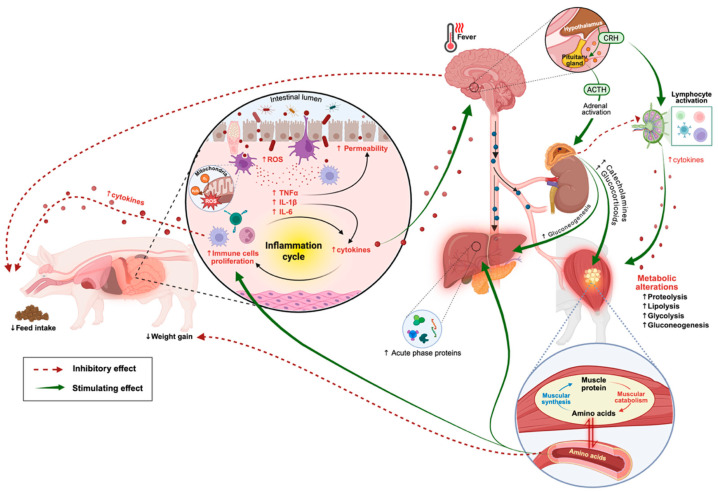
Representation of interactions between the immune response, neuroendocrine axis, and metabolism during a sanitary challenge with an enteric pathogen in pigs. The figure illustrates the systemic effects of an enteric infection in pigs, highlighting the immune, endocrine, and metabolic responses, as well as their complex interactions and consequences on feed intake and growth. The entry of an enteric pathogen (such as *Salmonella* or *E. coli*) activates immune-competent cells in the intestinal mucosa, leading to the production of reactive oxygen species (ROS) and increased intestinal permeability. This process induces the release of pro-inflammatory cytokines (TNF-α, IL-1β, IL-6), triggering the local inflammation cycle. The elevation (↑) of cytokines promotes the proliferation of immune cells and amplifies the inflammatory cycle. Pro-inflammatory cytokines reach the central nervous system and activate the hypothalamic–pituitary–adrenal (HPA) axis. The hypothalamus releases corticotropin-releasing hormone (CRH), which stimulates the anterior pituitary to secrete adrenocorticotropic hormone (ACTH), ultimately activating the adrenal glands. The adrenal cortex releases glucocorticoids (which modulate inflammation and promote metabolic adjustments during immune challenge), while the adrenal medulla secretes catecholamines (which rapidly mobilize energy substrates). In addition to stimulating lymphocyte activation (T and B cells), the HPA axis also contributes to fever development, which is triggered when pro-inflammatory cytokines stimulate the hypothalamus to produce prostaglandin E2 (PGE_2_). To meet the high energy demand imposed by inflammation, several metabolic pathways are activated, including proteolysis (↑ muscle protein degradation), lipolysis (↑ fat mobilization), glycolysis, and gluconeogenesis (↑ glucose availability). These alterations result in increased amino acid release, which is redirected toward glucose synthesis, acute-phase protein (APP) production, and inflammatory mediator synthesis. The liver responds to inflammation by increasing the production of APP, which supports immune modulation, nutrient transport, and host defense. Altogether, the activation of these systems leads to reduced (↓) feed intake and ↓ weight gain, as physiological priorities shift toward immune defense and the maintenance of body homeostasis.

Depending on the severity and nature of SC, the innate immune system may be insufficient to completely eliminate the infection, leading to the activation of the adaptive immune system. This process involves the proliferation of T and B lymphocytes [26], which demands AA (from the diet or body reserves; Figure 1) for their synthesis [34]. For instance, enhanced lymphocyte proliferation was observed in 49-day-old piglets challenged with *Salmonella Enteritidis*, 12 days after inoculation [35]. Although the development of the adaptive immune system can take several days to weeks, it establishes immunological memory, enabling pigs to better cope with prolonged or repeated exposure to SC [36].

Tissue injury and inflammation can also activate additional physiological mechanisms through the sympathetic nervous system and the hypothalamic–pituitary–adrenal (HPA) axis. Pro-inflammatory cytokines may stimulate the production of corticotropin-releasing hormone (CRH) in the central nervous system [37], leading to increased cortisol secretion. The increase in cortisol level can suppress anabolic hormones such as insulin and insulin-like growth factor 1 [38], thereby affecting protein metabolism and nutrient partitioning ([39]; Figure 1). For instance, Campos et al. [40] reported metabolic changes in growing pigs following a SC, such as increased plasma concentrations of glucose and non-esterified fatty acids, along with reduced postprandial concentrations of AA, such as Trp.

Finally, the presence of cytokines in the central nervous system can influence pig behavior and cognitive function [41], contributing to reduced feed intake and growth. These cytokines may stimulate the release of anorexigenic catecholaminergic neurotransmitters [42] or inhibit neuropeptide Y, a potent stimulator of appetite [43]. The reduction in voluntary feed intake observed in challenged pigs may also be attributed to the increase in signaling molecules such as prostaglandin E2 (PGE2), which impair gastrointestinal motility and delay gastric emptying [44,45], thereby decreasing appetite. In addition, PGE2 and cytokines such as interleukin (IL) 1β (IL-1β) stimulate leptin production by adipocytes, which induces an inflammatory state, reduces feed intake, and increases energy expenditure [46]. The magnitude of the feed intake reduction is influenced by the type, class, and severity (time and/or pathogen dosage) of the SC. At subclinical level, voluntary feed intake may decrease by up to 80% [47], while in a more severe SC, the voluntary feed intake can approach zero.

Furthermore, cytokines produced during the inflammatory response can inhibit muscle protein synthesis [48] and induce muscle protein degradation [49]. Consequently, there is an increased muscular catabolism and redistribution of nutrients (especially AA; [50]), and energy [51] from growth toward immune response [52]. This shift adversely affects protein deposition and compromises overall animal growth. For instance, piglets under a SC exhibited elevated plasma levels of alpha1-acid glycoprotein, a biomarker indicative of cytokine release, and showed a 28% reduction in protein deposition and a 20% decrease in nitrogen retention, compared to non-challenged counterparts [53,54].

## 3. Evidence from Experimental Models

In both domestic and laboratory animals, evidence of the physiological and metabolic changes associated with immune activation comes from experimental SC models. The most commonly observed outcomes in these models include reduced feed intake and protein synthesis, along with increased energy expenditure and body temperature [55]. However, the duration of these responses depends on the type and intensity of SC, as well as the pig’s ability to cope with the challenge by activating their immune system and eliminating the pathogens [56,57].

Several SC models have been described in the literature, including the inoculation of live microorganisms (such as *Escherichia coli* (ETEC), *Salmonella* spp., and *Lawsonia intracellularis*), the administration of chemical compounds like bacterial lipopolysaccharide (LPS), or models that mimic the deterioration of housing conditions. A detailed description of these SC models and the relevant response criteria in pigs for research applications was previously published [58,59]. Here, we evaluate the advantages and limitations of each model to help the reader identify which one most accurately reflects on-farm conditions.

LPS has been widely used as a SC model in farm animals [60]. LPS is a component of the outer membrane of Gram-negative bacteria that elicits a strong immune response [61]. It is well documented that LPS administration increases body temperature [62], reduces feed intake [63], alters the plasma concentration of acute phase proteins, activates the HPA axis [63], and inhibits the somatotropic axis [64]. Consequently, LPS impairs growth and contributes to economic losses [65]. Although the LPS model has contributed to the understanding of certain physiological and metabolic responses, this approach has limitations. The model usually induces only a short-term immune system response rather than the prolonged or chronic activation often observed under commercial conditions [48]. Such transient effects are partly due to the pigs’ ability to develop immune tolerance following repeated LPS administrations [65,66]. As a result, alternative SC models involving the oral inoculation of live pathogens have been proposed to better mimic real-world infectious challenges.

Enterotoxigenic *E. coli* (ETEC) is the primary causative agent of post-weaning diarrhea in nursery pigs, with a reported mortality rate reaching up to 30% [67]. In addition, the F4 (or K88) fimbrial antigen is the most frequently isolated in piglets and is associated with colibacillosis. Therefore, an oral inoculation with ETEC F4/K88 has been widely used as a SC model for post-weaning diarrhea [58,68]. *E. coli* adheres to the intestinal mucosa, disrupts intestinal morphology, characterized by reduced villus height and increased crypt depth, and impairs nutrient absorption, leading to diarrhea and reduced performance [69]. Other effects observed by ETEC include increased serum concentrations of urea [70], haptoglobin [71], pro-inflammatory cytokines (TNF-α and IL-6; [72]), fecal consistency score [58], and rectal temperature [17].

Although this model is widely used to elucidate the SC effect on pigs over a longer period (chronic response) compared to LPS, the ETEC impact on pig performance remains controversial in the literature, which represents a limitation. The age and weight of the pig at the time of challenge may influence the effectiveness of this SC model. The expression of ETEC receptors for fimbriae F4 and F18 is highest in the small intestine during the first three weeks of age. As a result, the higher infection rate of ETEC occurs mainly during the neonatal and weaning period [73]. After this period, the immune system matures and becomes fully developed between 6 and 8 weeks of age [74], leading to a lower density of ETEC receptors and increased immune competence. For this reason, another SC model using live pathogens for growing pigs was developed to evaluate the interactions between immune system activation and depressed growth: oral inoculation with *Salmonella* spp.

Salmonellosis is among the top 10 diseases affecting pigs during the growing and finishing phases [75]. It is estimated that a large proportion of the herd can be affected by *Salmonella* spp., with prevalence ranging from 25% and 50% [76,77]. The most commonly isolated is *Salmonella enterica* serovar Typhimurium, known for its high environmental persistence. Pigs challenged with ST typically exhibit clinical signs such as diarrhea, anorexia, and lethargy. In addition, ST triggers increased serum haptoglobin concentrations [78], rectal temperature, and upregulated synthesis of pro-inflammatory cytokines in the intestine [79,80], along with a general reduction in growth performance [15,78].

The inoculation dose of ST commonly ranges from 10^7^ to 10^9^ colony-forming units (CFU), given that pigs are able to shed high levels of the pathogen, between 10^5^ and 10^8^ CFU/g of feces. However, outcomes vary widely within this dosage range. Some studies report mild clinical effects [23,81,82,83]; while other studies have observed more pronounced responses [48,84], and even severe cases that exceeded the capacity for supportive treatment [85]. This variability represents a significant limitation of the ST challenge model for simulating sustained immune activation.

Due to the limitations associated with ST and ETEC and considering that pigs in conventional housing systems may be exposed to several pathogens simultaneously, Le Floc’h et al. [59] proposed an alternative challenge model based on deterioration of housing conditions. In this model, pigs are raised in facilities without disinfection and cleaning prior to or during their occupancy, thereby increasing microbial pressure in the environment. This continuous exposure activates the immune system and impairs growth performance, yet without inducing overt clinical signs such as prostration, dehydration, vomiting, and complete anorexia.

Typical responses in pigs kept under poor hygiene conditions include elevated serum haptoglobin concentrations [86], reduced villus height and increased intestinal crypt depth [87], decreased feed intake and weight gain, and low feed efficiency [13,16,59,88]. This SC model can be applied to a large group of pigs and is less costly than the other models. However, its major drawbacks lie in the difficulty of standardizing the degree of housing deterioration and identifying the specific agents responsible for the immune activation, which compromises the reproducibility across time and laboratories.

Each challenge model discussed here is important for elucidating the interactions between pathogens and animal homeostasis; however, taken together, each approach has inherent advantages and limitations (Table 1). Therefore, the selection of an appropriate SC model should consider factors such as the research objectives, biosecurity requirements, pig production phase, type of immune response (acute or chronic), and feed additive under evaluation, among others [58,59].

## 4. Roles of Functional Amino Acids: A Focus on Met, Trp, and Thr

Pigs raised in conventional commercial production systems are routinely exposed to SC, mainly through the inhalation or ingestion of antigens and pathogens, which may induce an immune response. Under such conditions, maintenance nutrient requirements may increase by 5–7% in monogastric animals [89]. Immune system activation affects several metabolic processes involving AA and proteins, not only due to their role as building blocks for protein synthesis but also because of their involvement in immune and inflammatory responses [90]. Therefore, AA becomes a strategic target for dietary adjustments aimed at modulating key metabolic pathways to support animal health and optimize growth performance under immunological stress [9,44,90,91].

However, the effects of SC on pig metabolism and nutritional requirements are rarely considered when formulating diets. This raises the question of whether the current AA recommendations for growing-finishing pigs, estimated to maximize the growth performance of healthy pigs (NRC, [21]), are truly adequate for pigs under immunological stress. Therefore, by overlooking the metabolic roles of AA during immune activation, conventional nutritional programs may present limitations when applied to commercial production settings. In such scenarios, increasing the dietary levels of specific AA may serve as a strategy to restore the balance between immune function and growth performance.

Additionally, when AA in free form are supplemented in the diet, they appear in peripheral plasma more rapidly than those derived from intact dietary proteins [92]. Yen et al. [93] observed that the peak concentrations of lysine (Lys) and Thr in arterial and portal plasma of growing pigs occurred approximately one hour after the ingestion of free AA in the diet, whereas AA originating from intact proteins reached peak levels after 2.5 h. This difference in absorption kinetics provided a physiological basis for using different forms of AA as a strategy for preventive or therapeutic nutritional intervention through the diet [9].

Certain AA have been adopted as nutritional strategies to attenuate the negative effects of SC on pigs’ growth due to their roles in modulating immune responses. Among them, Met has received particular attention for its involvement in immune function [94,95] and its essential role in the redox balance [96]. Oxidative stress arises from an imbalance in the organism between the endogenous production of reactive oxygen species (ROS) and antioxidant defenses [97]. Some immune cells, such as macrophages, produce ROS as part of their cytotoxic activity, which may, in turn, exacerbate oxidative stress and overwhelm the antioxidant defenses [44]. To improve the antioxidant capacity under an immune system activation, a considerable proportion of sulfur AA, such as Met, is diverted from protein synthesis toward the production of non-protein compounds, such as glutathione [96]. For instance, Le Floc’h et al. [59] observed a decrease in plasma concentration of sulfur AA and glutathione in weaned piglets subjected to SC. Similarly, Rakhshandeh and de Lange [98] reported increased blood concentrations of glutathione in challenged growing-finishing pigs, attributed to the enhanced conversion of Met and cysteine (Cys). These findings suggest that Met supplementation can contribute to strengthening antioxidant defenses by supporting glutathione synthesis and aiding in the neutralization of ROS generated by activated immune cells.

Another important AA for SC pigs is Trp. During the immune system activation, gamma interferon activates the enzyme indoleamine 2,3-dioxygenase (IDO), whose function is the catabolism of Trp into kynurenine [12,99]. The IDO activation and the Trp metabolites production regulate the T cell proliferation, coordinating the long-lasting immune activation [100] and immunotolerance [101]. Additionally, oxidative stress has been shown to reduce plasma Trp concentration while increasing plasma kynurenine levels [102]. Such modifications are expected to affect Trp availability for growth. Therefore, dietary Trp supplementation may be beneficial for pigs under SC [9,87]. For instance, dietary supplementation with an additional 1 g Trp/kg above standard requirements improved growth performance in weaned pigs genetically predisposed to *E. coli* compared to non-susceptible pigs [69].

**Figure 2:**
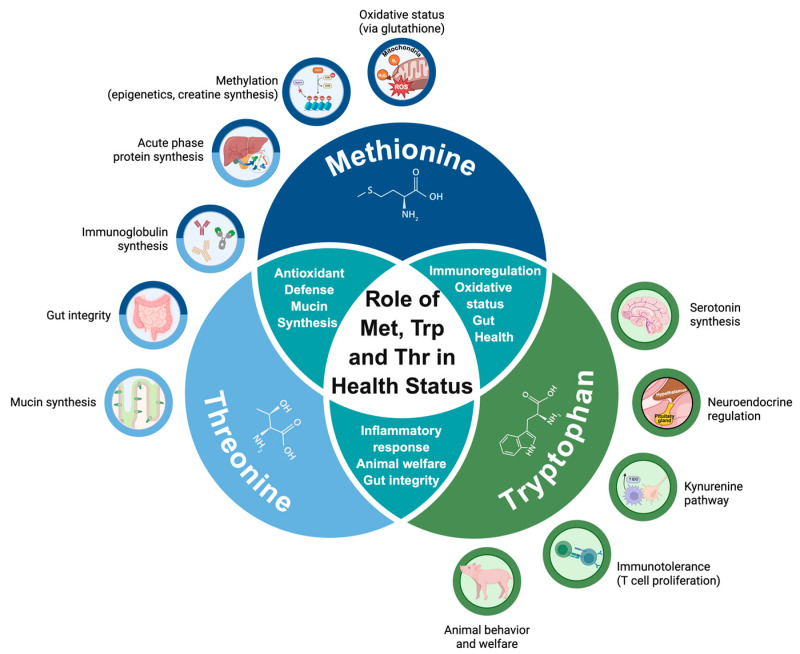
General roles of methionine (Met), tryptophan (Trp), and threonine (Thr) in swine health. This overview highlights their key physiological and immune functions, emphasizing contributions to antioxidant defense, gut integrity, and animal welfare.

Finally, Thr is another functional AA that plays an important role in supporting pigs under SC caused by enteric pathogens. Typically, enteric pathogens activate the innate immune system and compromise gut barrier integrity, leading to leaky gut and diarrhea [103,104]. In this context, an adequate Thr supply can contribute to the maintenance of intestinal functionality [105] by reducing inflammation and preventing pathogen translocation [106]. Thr is the major constituent of mucus glycoproteins (10–13%; [107]) and immunoglobulin A (7–11%; [56]), both of which are essential for mucosal defense. Therefore, Thr may benefit pigs by reducing the gut inflammatory response [69] by enhancing mucus synthesis and the binding of bacteria to the mucosal surface [108]. Experimental evidence supports this role: in an in vitro model, Thr-deprived intestinal cells exposed to an *E. coli* showed a compensatory increase in the expression of IL-8, mucin 2, and immunoglobulin A mRNA, which was suppressed upon Thr supplementation [109]. In vivo studies also demonstrate performance benefits in pigs under a SC with dietary Thr supplementation [15,18]. Figure 2 highlights the principal functions of methionine (Met), tryptophan (Trp), and threonine in supporting swine health, as identified in our review.

## 5. Dietary Met, Trp, and Thr Supplementation Mitigates Performance Loss and Behavioral Alterations for Sanitary-Stressed Group-Housed Pigs

It is well established that nutritional status and the immune system are interconnected. Therefore, dietary supplementation with Met, Trp, and Thr may help offset the reduced nutrient availability and feed intake observed during a SC. Considerable variability has been observed among studies about the impact of Met, Trp, and Thr supplementation, including differences in challenge severity, animal characteristics, and methodological approaches (Table 2). Therefore, direct extrapolation of findings from controlled experimental systems to commercial production should be approached with caution for practical application. Furthermore, to the best of our knowledge, few studies have investigated the effects of dietary AA supplementation on growing pigs over an extended period (more than two weeks) under SC conditions [13,22,23]. Moreover, it remains unclear whether this nutritional strategy can be extrapolated to a commercial system, where pigs are group-housed and constantly exposed to multiple antigens, without severe clinical signs of disease or mortality [13]. Such a condition may require the simultaneous supplementation of multiple AA.

Furthermore, a few studies have assessed the impact of AA supplementation on the efficiency of nutrient and energy utilization for lean deposition. The available data in the literature regarding the effect of SC on body composition are from slaughterhouse measurements [13], which allow only a single measurement throughout the experimental period. In this context, the use of dual-energy X-ray absorptiometry (DXA) is advantageous, as it allows repeated, non-invasive measurements in the same animal and with high precision for estimating body composition. In a recent study conducted in our laboratory using DXA, the supplementation of Met + Cys, Trp, and Thr at levels 20% above the NRC [21] requirements improved growth performance, mitigated immune activation, and enhanced protein deposition and nitrogen utilization efficiency in ST-challenged growing group-housed pigs raised under poor housing conditions [23].

In line with these findings, previous studies have reported beneficial effects of AA supplementation in nursery piglets challenged with ST, whether provided exclusively as a preventive measure [121] or as a combination of preventive and curative strategies [119]. However, the effectiveness of a long-term supplementation strategy—starting in the nursery phase (preventive) and continuing through a health challenge in the growing phase (curative) has not been thoroughly explored yet.

In this context, our recent findings [22] demonstrated that curative supplementation with additional Met + Cys, Trp, and Thr (20% above the NRC [21]) during a multifactorial health challenge (including batch mixing, poor housing conditions, and ST infection) improved growth performance, nitrogen retention, and protein accretion for group-housed growing pigs. Moreover, both curative and continuous supplementation strategies led to heavier pigs with increased body protein mass by the end of the finishing phase.

Dietary protein deficiencies or AA imbalances may affect animal behavior in intensive production systems. Relative shortages of one or more AA have been associated with increase exploratory activity [122] and aggressive behaviors toward the pen [123]. For instance, the effect of dietary Trp on feeding and aggressive behavior has been associated with its role as a precursor of serotonin (5-hydroxytryptamine; 5-HT). Reduced stimulation of central 5-HT receptors lowers the production of orexigenic factors, thereby decreasing feed intake in pigs [124]. Additionally, lower levels of 5-HT have been associated with aggressive behavior, such as tail biting behavior in piglets [125]. Therefore, deficient dietary AA levels may impair pigs’ ability to cope with stressors in their environment.

Sanitary challenges can also contribute to AA imbalance by redirecting AA, such as Trp, towards immune system activation. This metabolic shift may lead to behavior changes in pigs [126]. For instance, weaned piglets housed in SC spent more time with exploratory behavior than with voluntary feed intake, which accounted for approximately 10% of the observed reduction in feed intake [127]. These modifications in feeding and exploratory behavior may be related to the feed aversion developed by pigs due to their association between feed ingestion and post-ingestion effects, such as abdominal discomfort [128].

Accordingly, the dietary supplementation of specific AA, such as Met, Trp, and Thr, may help mitigate behavioral disturbances. Growing pigs under SC exhibited increased competition at the feeder (+25%) and a higher incidence of stereotyped and aggressive behaviors, including ear and tail biting [126]. However, dietary supplementation of Met, Trp, and Thr 20% above the NRC [21] recommendations reduced ear biting frequency by 16% and decreased mounting behavior in growing pigs under SC [126]. The reduction in agonistic behavior may be associated with meeting the requirements of these AA to support the immune system, thereby decreasing the exploratory chewing behavior towards pen mates. This behavior improvement was also associated with enhanced performance, including greater average daily gain and gain-to-feed ratios [13].

In our recent findings, pigs housed in good condition when fed dietary functional AA supplementation (Met, Trp, and Thr 20% above the NRC; [21]) had lower total intake and shorter meal duration than those fed the control diet (without additional AA supplementation). However, pigs under SC fed the control or AA functional supplementation diet had no differences for both variables [24]. Feeding pigs *ad libitum* with greater amounts of crystalline AA without a SC may trigger consequences such as impair feeding patterns due to the postabsorptive physiological response to an imbalance of essential AA. It seems that AA requirements and voluntary feed intake are dependent on sanitary conditions. Nevertheless, information on the behavior of growing exposed to SC remains limited [127,128,129]. Studies recording feeding and aggressive behaviors can serve as a valuable tool to detect and better understand the relationship between SC, physiological responses, and nutritional requirements of SC pigs.

## 6. Integrating Functional Amino Acids and Precision Feeding to Support Pig Health Under Sanitary Stress

The practical application of phase feeding programs involves feeding pigs a unique diet formulated to meet the requirements of the most demanding pigs, aiming to maximize weight and lean tissue gain [130]. This approach assumes that the population’s growth response is equal for all pigs [131]. However, pigs of the same age often exhibit substantial inter-animal variation, which increases the variability in growth performance and reduces economic returns.

**Figure 3:**
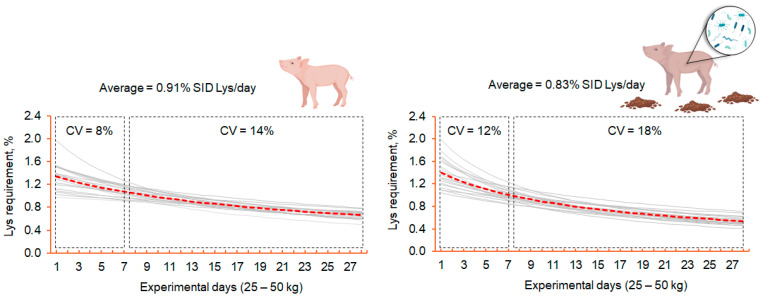
Predicted average (**---**) and individual (▬) Lysine (Lys) requirements of growing pigs throughout the 28-day experimental period in good sanitary conditions and poor sanitary conditions. CV: Coefficient of variation (%); Lys: Lysine; SID: Standard ileal digestible.

Moreover, individual pigs’ nutrient requirements, particularly AA, vary over time and can be influenced by several factors that contribute to differences in individual responses [130]. One such factor is exposure to environmental stressors [4,132], as individuals within a population may respond differently to environmental stimuli. Challenging sanitary conditions can impair intestinal AA absorption, increase endogenous protein losses in the gut, and promote AA oxidation to support immune response [4]. These physiological changes reduce growth performance and alter AA requirements. Specifically, SC not only impairs performance but also increases variability among pigs in terms of growth, body composition, and Lys requirements (Figure 3, [133]).

The extent to which immune stimulation impacts variability depends, among other things, on pigs’ ability to cope with a SC, which may be affected by body weight and body composition. According to resource allocation theory [134], a higher proportion of lean body mass is associated with greater capacity to respond to immune activation, as animals can redirect nutrients and mobilize body protein reserves to support immune response [135]. Therefore, heavier pigs may have a better coping ability than lighter counterparts.

Additionally, lighter pigs have lower feed intake and limited physical capacity to ingest sufficient nutrients compared to heavy pigs [136,137], which may increase their susceptibility to SC [138]. In addition, lighter pigs are more prone to being affected by stressors [139], a condition that has been associated with nutrient metabolism and increased intestinal inflammation. Therefore, the nutrient requirements, especially for AA, of the lightest pigs under a SC may vary greatly from those of heavier pigs. As a result, the dietary AA supplementation of Met, Trp, and Thr as a nutritional strategy to improve the growth performance of pigs under a SC has different effects on pigs with different body weight categories [24]. Since the beneficial effects of these AA are mainly associated with improved intestinal mucosa integrity, antioxidant defense, and immune molecule synthesis [9], this impact may be more pronounced in pigs with a higher susceptibility to stressors.

Recent advances, such as individual precision feeding (IPF), have been developed to manage variability in nutrient requirements by delivering customized diets that meet the specific daily needs of each pig [140]. Research indicates that IPF can substantially decrease lysine intake (by approximately 27%) and reduce nitrogen excretion (by around 30%) without negatively affecting growth performance [141,142]. These results highlight the potential of the IPF as a strategy to improve nutrient utilization, particularly in pigs facing immune challenges. Regarding the beneficial effects of function AA, its combination with IPF could increase the performance and the nutrient efficiency of immune-challenged pigs. However, further investigation is still required.

## 7. Future Perspectives

Under SC conditions, pigs exhibit physiology (e.g., increased rectal temperature and altered fecal score), behavior (e.g., deviation in feeding pattern), and metabolic (e.g., altered protein and energy metabolism) changes as adaptive mechanisms to reestablish homeostasis and maintain growth. However, depending on the pathogen involved (its type, pathogenicity, and duration of exposure), these responses may not be enough, resulting in negative impacts on pig health, welfare, and performance.

These adverse outcomes may be exacerbated in commercial production systems, since pigs are typically fed diets formulated to support their genetic growth potential under ideal conditions, without accounting for environment interaction and the variability between individual responses. Consequently, current feed formulation, feeding system, and genetic selection for high feed efficiency may inadvertently increase pigs’ susceptibility to SC under intensive production systems. This highlights the need for alternative strategies to mitigate health-related setbacks while addressing other ongoing challenges, such as environmental sustainability and rising feed costs—particularly in the context of increasing global pork demand. Thus, the supplementation of AA has gained attention as a potential strategy, given its involvement in modulating the immune system [9,10] and regulation of intestinal tight junction [143].

Our objective with the current review was to provide an in-depth discussion on the interactions between nutrition, pigs’ health status, and immune response, emphasizing how AA supplementation (in particular, Met, Trp, and Thr) can benefit pigs under SC conditions. These AA were selected based on the growing body of evidence highlighting their roles in immune modulation, oxidative stress reduction, and maintenance of gut integrity in pigs exposed to such challenges. In addition, Met, Trp, and Thr supplementation are more frequently used in commercial production systems due to their cost-effectiveness in feed formulation when compared to other AA. However, important new possibilities remain for future research. Future studies should explore supplementing functional AA alongside probiotics, prebiotics, organic acids, or phytogenics. For instance, early evidence indicates that a blend of functional AA and grape polyphenols improves the pig capacity to cope with an inflammatory challenge caused by poor hygiene of housing conditions [86]. Another important aspect is to obtain refined dose–response and ratio strategies. Although Met, Trp, and Thr at 120% of NRC recommendations have shown benefits, optimal dosing, balance, and interaction effects (including total protein context) remain underexplored [144]. Future research should map dose–response curves and identify ideal amino acid ratios for health and performance.

There is a need to move toward better elucidation regarding the molecular and metabolic mechanisms by which these AA regulate stress resilience and immune function under SC. This molecular approach could reveal key pathways such as Nrf2 signaling, mucin synthesis, and kynurenine-mediated immunomodulation. Furthermore, while Thr and Trp are known to influence gut microbiota toward beneficial profiles, deeper metagenomic, metabolomic, and mycobiome studies are required to establish causality and understand host–microbiome interplay [144]. Finally, given the individual variation in AA requirement during stress, dynamic and IPF systems that tailor AA supplementation in real time could improve pig resilience, reduce nitrogen excretion, and enhance resource efficiency.

Therefore, advancing these perspectives, through synergy with novel feed additives, precision nutrition, mechanistic insights, microbiome integration, and commercial validation, will deepen our understanding and application of functional AA strategies to support pig health and performance under sanitary stress.

## 8. Conclusions

Pigs’ responses to stressful situations include behavioral, physiological, and metabolic defense mechanisms aimed at re-establishing homeostasis and survival in a specific environment. Due to increased susceptibility of modern genotypes to environmental challenges, health-related issues have become especially critical in the current intensive production systems. Dietary supplementation with Met, Trp, and Thr enhances the immune response and, thus, is a promising strategy to minimize the impact of SC on pig performance, health status, and welfare. Given the increased variability in nutrient requirements among pigs under stress conditions, it is essential to implement innovative technologies that enable real-time, individualized daily feeding. Such precision nutrition approaches contribute to more sustainable production systems by optimizing resource use, reducing nutrient excretion, and minimizing the environmental footprint of pig farming.

## Figures and Tables

**Table 1:** Comparison of sanitary challenge models used in pig studies.

Challenge Model	Advantages	Disadvantages
Specific pathogen challenge	- Mimics real infections observed under field conditions - Allows investigation of pathogen–host interactions	- Requires high biosafety facilities - Higher biosecurity risk - Lower reproducibility between studies
Poor hygiene environment	- Simulates multifactorial challenges similar to commercial settings - Low operational cost	- High variability between trials - Limited control over challenge intensity
LPS injection (acute inflammatory model)	- Highly controlled and reproducible - Induces rapid systemic inflammatory response - Useful for mechanistic studies	- Does not mimic natural infections - Transient and artificial effect - Limited application to chronic stress models

LPS: Lipopolysaccharide.

**Table 2:** Summary of experimental challenge models and pig responses to dietary Met, Trp, and/or Thr supplementation.

Author	Phase	Type of Challenge	AA Supplementation	ADG and ADFI Compared to Control	General Observations
Trevisi et al. [69]	Nursery	ETEC	Trp	↑ 22% ADG ↑ 8% ADFI	↓ rectal temperature ↓ ETEC count ↑ [serum IgA]
Le Floc’h et al. [99]	Nursery	Poor sanitary condition	Trp	↑ 9% ADG ↑ 6% ADFI	↑ 3% feed efficiency ↓ [IFN-α]
Mao et al. [110]	Nursery	*Pseudorabies* live vaccine	Thr	↑ 7% ADG ↑ 8% ADFI	↑ 8% feed efficiency ↑ [serum IgA, IgM, IgG]
Ren et al. [111]	Nursery	ETEC	Thr	↑ 7% ADG ↑ 1% ADFI	↑ Lymphocyte proliferation ↑ [serum IgA, IgG]
Capozzalo et al. [112]	Nursery	*Escherichia coli*	Trp	↑ 16% ADG ↑ 14% ADFI	↑ 12% BW ↑ 4% feed efficiency ↓ [urea]
Jayaraman et al. [18]	Nursery	Poor sanitary condition	Thr	↓ 21% ADG ↓ 25% ADFI	↑ 9% feed efficiency
Pinheiro et al. [113]	Nursery	*Mycoplasma hyopneumoniae* vaccine	Met	↑ 7% ADG ↑ 1% ADFI	↑ 6% feed efficiency ↑ 7% protein deposition ↓ 70% fat deposition
Xu et al. [114]	Growing	Ovalbumin	Trp and Thr	↑ 10% ADG ↑ 8% ADFI	↑ 4% feed efficiency ↑ Lymphocyte proliferation ↓ cellular damage
Van der Meer et al. [13]	Growing	Poor sanitary condition	Trp, Thr, and Met	↑ 4% ADG ↑ 2% ADFI	↓ [leukocytes]
Jayaraman et al. [115]	Nursery	*Escherichia coli*	Trp	↑ 18% ADG ↑ 10% ADFI	↑ 6% feed efficiency ↓ plasma urea
Kahindi et al. [116]	Nursery	Poor sanitary condition	Met	↑ 10% ADG ↑ 8% ADFI	↑ 2% feed efficiency ↑ 4% BW
Kahindi et al. [19]	Nursery	*Escherichia coli*	Met	↑ 7% ADG ↑ 7% ADFI	↑ 6% feed efficiency ↓ plasma TNF- α
Wellingtion et al. [15]	Growing	*Salmonella* Typhimurium	Thr	↑ 12% ADG ↓ 6% ADFI	↑ 17% feed efficiency ↓ *Salmonella* Typhimurium in the cecum and colon
Van der Meer et al. [117]	Nursery	Poor sanitary condition	Trp, Thr and Met	↑ 14% ADG ↓ 5% ADFI	↑ 11% feed efficiency ↑ [serum IgG] ↓ [haptoglobin]
Sterndale et al. [118]	Nursery	ETEC	Trp	↑ 18% ADG ↑ 6% ADFI	↑ [cortisol] ↓ [plasma Trp]
Rodrigues et al. [14]	Nursery	*Salmonella* Typhimurium	Trp, Thr, and Met	↑ 35% ADG ↓ 8% ADFI	↑ 6% BW ↑ [albumin] ↓ [superoxide dismutase]
Rodrigues et al. [119]	Nursery	*Salmonella* Typhimurium	Trp, Thr, and Met	↑ 54% ADG ↑ 1% ADFI	↑ 54% feed efficiency ↓ *Salmonella* Typhimurium translocation (from intestine to spleen) ↑ [albumin] ↓ [haptoglobin]
Valini et al. [24]	Growing	*Salmonella* Typhimurium + poor sanitary condition + animal group mixing	Trp, Thr, and Met	↑ 14% ADG ↑ 4% ADFI	↑ 13% feed efficiency ↑ 7% BW ↑ 14% protein deposition ↑ [albumin] ↓ [urea and creatinine]
França et al. [22]	Growing	*Salmonella* Typhimurium + poor sanitary condition	Trp, Thr, and Met	↑ 29% ADG ↑ 15% ADFI	↑ 17% feed efficiency ↑ 12% BW ↑ 25% protein deposition ↑ [albumin and glucose] ↓ [urea]
Gonçalves et al. [120]	Nursery	Poor sanitary condition	Trp, Thr, and Met	↑ 23% ADG ↑ 21% ADFI	↑ 13% BW ↓ [urea and creatinine]

ADFI, Average daily feed intake; ADG, Average daily gain; BW, body weight; ETEC, Enterotoxigenic *Escherichia coli*; IFN-α, Interferon-alpha; LPS, Lipopolysaccharide; Met, Methionine; Thr, Threonine; Trp, Tryptophan.

## Data Availability

No new data were created or analyzed in this study. Data sharing is not applicable to this article.
